# Efficacy of epidural blood patch with fibrin glue additive in refractory headache due to intracranial hypotension: preliminary report

**DOI:** 10.1186/s40064-016-1975-1

**Published:** 2016-03-11

**Authors:** Justin J. Elwood, Misha Dewan, Jolene M. Smith, Bahram Mokri, William D. Mauck, Jason S. Eldrige

**Affiliations:** Department of Anesthesiology, Mayo Clinic, 200 First St SW, Rochester, MN 55905 USA; S.E. PA Pain Management, 721 Dresher Road, Suite 2500, Horsham, PA 19044 USA; Pain Specialists of Iowa, 12499 University Ave, Suite 280, Clive, IA 50325 USA; Department of Neurology, Mayo Clinic, 200 First St SW, Rochester, MN 55905 USA

**Keywords:** Orthostatic headache, Spontaneous intracranial hypotension, SIH, Blood patch, Fibrin glue

## Abstract

**Background:**

Injection of fibrin glue mixed with blood into the epidural space to reliably and effectively treat medically refractory orthostatic headache caused by spinal cerebrospinal fluid (CSF) leaks and subsequent intracranial hypotension has recently been described. The study described in this article utilizes an analogous technique to gauge the therapeutic reproducibility of this novel technique.

**Methods:**

Eight patients with medically refractory headache resulting from intracranial hypotension caused by spinal CSF leaks received epidural injections of combined fibrin glue, autologous blood, and Isovue contrast at the L1—2 vertebral level using intermittent fluoroscopic guidance. Pre-procedure, 1-week post-procedure, and 3-month post-procedure headache pain scores were collected and used for comparison.

**Results:**

Three out of 8 patients reported relief at 1 week, although 1 of these 3 patients had returned to their baseline pain intensity at 3 months. Four patients reported no change at 1 week, though 2 of these patients had reduction of their chronic headache pain at 3 months. A single patient reported increased pain 1 week after the procedure, which persisted at 12 weeks. Overall, 4 out of the 8 patients had decreased pain scores at 3-month follow-up.

**Conclusions:**

We did not achieve a similar frequency of headache resolution as reported in prior original studies. However, a subset of patients did appear to receive substantial benefit from the combined fibrin glue-blood patching procedure. This technique may prove to be useful in medically refractory cases, including those patients who continue to have symptoms despite the prior administration of conventional epidural blood patches.

## Background

Headache associated with spontaneous intracranial hypotension (SIH) is often although not always orthostatic (provoked or aggravated in upright position and decreased or relieved in recumbency). The diagnosis is made based on the synthesis of patient history, clinical findings, cerebrospinal fluid (CSF) examination, and imaging studies. These headaches, which are now well defined in the scientific literature, can be frontal, occipital, frontooccipital or holocephalic in anatomic location (Scott and Davenport 2014; Mokri 2014). They are often pressure-like, occasionally throbbing, and typically bilateral. With chronicity the orthostatic features of the headache may dampen and they may transform into chronic daily headaches. In some cases the headaches are exertional or may even have thunderclap onset before orthostatic features arise (Spears 2014). Other symptoms that are frequently encountered with the headache include neck or interscapular pain, cochleovestibular symptoms (tinnitus, changes in hearing, dizziness), nausea, emesis, diplopia, mild unsteadiness, memory deficiencies, personality changes and a variety of less common manifestations; giving rise to a broad spectrum of clinical presentation that can be quite different from classic post dural puncture headaches (Scott and Davenport 2014; Mokri 2014; Spears 2014). CSF opening pressure is often low or borderline but may also be normal. Head magnetic resonance imaging (MRI) typically shows diffuse pachymeningeal gadolinium enhancement without leptomeningeal enhancement (Mokri 2014; Mitsikostas et al. 2015). Additionally, imaging often reveals evidence of sinking of the brain (descent of cerebellar tonsils and brainstem, crowding of the posterior fossa), subdural fluid collections and pituitary engorgement with enlargement. It is now recognized that nearly all cases of SIH result from spontaneous CSF leaks, which occur at the level of the spine (Inamasu and Guiot 2006). Spontaneous CSF leak at the level of skull base does occur but only rarely. Evidence of spinal CSF leak may be shown by spine MRI, computed tomography myelography (CTM), or both. In select cases with slow CSF leaks, gadolinium myelography may be helpful (GdM; spine MRI after intrathecal injection of gadolinium). Radioisotope cisternography may also show the site of CSF leakage, but is considered to be less sensitive than CTM. The precise etiology of spontaneous (non-traumatic) CSF leaks is often unclear, but it is now increasingly recognized that some of these patients show evidence of disorder of connective tissue matrix leading to dural weakness that can predispose to spontaneous leaks (Mokri 2014; Schrijver et al. 2002).

Epidural blood patch (EBP) has emerged as the treatment of the choice for patients who fail initial conservative management. The mechanism of action for blood patching is twofold, with both immediate and delayed effects. The immediate effect results from tamponade of the dural sac (by injected epidural blood), leading to translocation of axial CSF to the intracranial compartment and subsequent increased CSF pressure. Delayed effects are attributed to actual sealing of the leakage site, which allows the intrathecal space to be reconstituted with normal volume as CSF is naturally regenerated and recirculated. Previous studies have pointed to success rates ranging from 36 to 85 % after a single epidural blood patch for SIH headaches (Sencakova et al. 2001; Gaukroger and Brownridge 1987; Berroir et al. 2004). Those who do not respond to epidural blood patch are typically considered to have refractory headaches, although the exact definition of refractory headache has not yet been agreed upon by the medical community (Lampl et al. 2014). Fibrin glue has also been used as an alternative to blood, particularly in cases where the use of autologous blood has not been feasible, when a smaller volume of epidural injection is preferred, or when the leak has persisted despite trials of multiple EBPs. Several studies and reports have confirmed efficacy of epidural fibrin glue injection, without the addition of additional blood (Schievink 2008; Patel et al. 1996).

Two studies (Franzini et al. 2010, 2013) have proposed epidural injections of fibrin glue mixed with blood as an effective method to reliably treat medically refractory orthostatic headaches caused by intracranial hypotension. In a study published in 2010 (Franzini et al. 2010) involving 28 patients, at 3 months follow-up 70 % were headache free and 22 % complained of only sporadic headaches. In a study published in 2013 (Franzini et al. 2013), there were 80 patients with 3 months follow-up. After a single procedure, 87 % reported complete resolution of their symptoms. The non-responders then received a second epidural patch, and a total of 90 % of patients reported complete resolution of symptoms at 3 months. The aim of our study was to assess the overall efficacy of EBP plus fibrin glue additive and attempt to reproduce the results of these prior studies.

## Methods

**Fig. 1:**
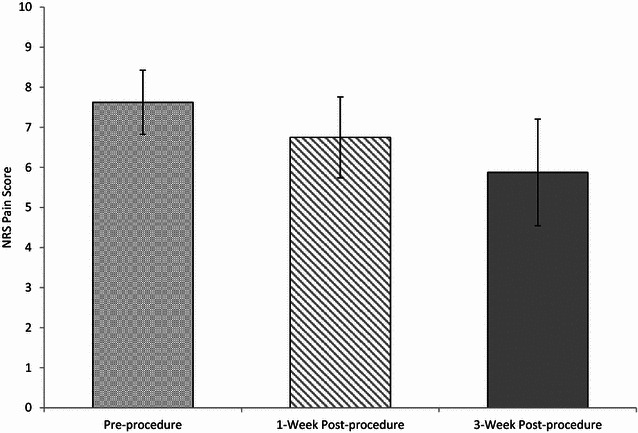
Mean pre-/post-procedure pain scores. Shown are mean ± standard error of the mean (SEM) NRS pain scores at the noted time points. Statistical analysis (two-tailed Student’s *t* test) revealed no significant difference in mean pain score between pre-procedure and 1-week post-procedure or between pre-procedure and 3-week post-procedure. Median pain scores for each group were 8.5, 7.5, and 7 for pre-procedure, 1-week post-procedure, and 3-week post-procedure, respectively

Following IRB approval from our institution, we enrolled 8 patients—5 females and 3 males—with refractory headaches related to SIH or dural puncture and treated them with epidural injections of blood with fibrin glue at the L1 spine level. Refractory headaches were defined as those with symptoms that had failed to resolve with at least one prior epidural blood patch performed using traditional technique with fluoroscopic guidance. Each patient also had neurologic evaluation by a neurologist prior to the epidural injection with confirmation of the diagnosis of refractory headache caused by intracranial hypotension. Using the numeric rating scale (NRS), pre-procedure and 1 week post-procedure headache pain scores were collected and available to review via electronic medical records. The patients were contacted via telephone at 3-month post-procedure and headache pain scores were recorded at that time. Patients were also questioned about duration of pain relief and functional improvement after receiving the fibrin glue blood patch. To further assess patient satisfaction, they were also asked if they would recommend this procedure to others or if they would be willing to undergo a repeat procedure. Mean and median NRS score were graphed to compare changes (see Fig. 1).

The fibrin glue sealant, Baxter Tisseel©, was used due to its similarity to the fibrin product used in 2 previous publications (Franzini et al. 2010, 2013). Our protocol also required fluoroscopically accessing the L1—2 epidural space with an 18 gauge Tuohy needle (using a midline, interlaminar approach). Needle placement was then confirmed with Isovue contrast administration, in both the lateral and anterior-posterior fluoroscopic views. Next, 10 ml of autologous blood was sterilely withdrawn from the patient and briefly set aside after mixing the obtained blood with 3 ml of additional Isovue contrast. The Tisseel© syringe was attached to extension tubing connected to the Tuohy epidural needle hub, and fibrin glue was then injected rapidly into the posterior epidural space until a pressure paresthesia of at least 2–3 out of 10 intensity or greater was experienced by the patient. Note that pressure paresthesia was defined as significant pressure sensations felt by the patient in the head, back, buttock or legs (whether unilateral or bilateral). Up to 10 ml of fibrin glue injectate was available to inject (the full capacity of the Tisseel© syringe), although the actual fibrin glue volume utilized was dependent upon the onset and intensity of patient paresthesia response alone (i.e., fibrin glue was injected until the patient experienced this paresthesia response). Thereafter, the previously prepared blood-Isovue mixture was injected immediately afterward through a new connection tube. Only enough blood mixture was injected to clear the epidural needle from fibrin glue (less than 1 cc), while immediately ceasing further injection until the patient’s pressure paresthesia had fully resolved to its pre-procedural baseline. Once the patient’s paresthesia had fully resolved, additional blood-Isovue admixture was injected until the patient again experienced a pressure paresthesia of at least 2–3 out of 10 intensity. The epidural needle was then gently flushed with saline or 1 % lidocaine as it was withdrawn from the epidural space and fully removed from the skin. Patients were asked to remain supine in our recovery area for about 30 min post-procedurally to allow further stabilization of the injectate in the epidural space.

## Results

Each of our 8 patients reported pain scores using the NRS system at three time points (Table 1). Three patients reported relief at 1 week, however only 1 patient in that group experienced greater than 50 % relief. Four patients reported no change at 1 week, though 2 of these patients had reduction of their chronic headache pain when further assessed at 3 months. A single patient reported a 3 point increase in pain after the procedure, which persisted at the 12-week follow-up. Of the patients who had immediate post-procedure relief (3), only 1 patient had worsening of pain back that returned to baseline intensity at 3 months (others maintained benefit or had further pain attenuation at 3 months). Also at 3 months, 50 % of the patients who did not find immediate relief continued to experience the same level of discomfort while the other 50 % reported a significant reduction or even resolution of their symptoms. Two patients underwent a second fibrin glue blood patch procedure.

**Table 1:** Patient information, history, and pre-/post-procedure pain scores

Case number	Sex	Diagnosis	Number of fibrin glue-blood patch procedures	Pre-procedure pain score^a^	1-Week post-procedure pain score^a^	3-Month post-procedure pain score^a^
1	F	Orthostatic, exertional, and valsalva headache	2	6	9	9
2	F	Intracranial hypotension headache	1	10	10	10
3	F	Intracranial hypotension headache	1	9	6	6
4	M	Spontaneous CSF leak with headache	2	9	9	9
5	M	Intracranial hypotension headache	1	8	3	8
6	F	Intracranial hypotension headache	1	9	9	3
7	M	Intracranial hypotension headache	1	7	5	2
8	F	Intracranial hypotension headache	1	3	3	0

*CSF* cerebrospinal fluid, *F* female, *M* male

^a^Pain scores are on a scale from 1 to 10

In summary, overall there was a slight trend downwards in mean and median pain scores 1 week post-procedure as compared to pre-procedural baseline (Fig. 1). At 3 months post-procedure, 4 out of 8 patients had decreased pain scores.

## Discussion

The purpose of our study was to evaluate the efficacy of epidural injection of combined fibrin glue and blood to treat medically refractory low CSF pressure, low volume headaches. In contrast to the previous original reports (Franzini et al. 2010, 2013) we encountered several challenges with the procedure itself and also did not achieve similar results, at least in terms of frequency of headache resolution.

It has been theorized by one group that negative pressure in the epidural space occurs with standing, resulting in aspiration of CSF into the epidural veins and back to the vena cava system which facilitates a physiologic state of prolonged intracranial hypotension (Franzini et al. 2010, 2013). Similarly unsubstantiated, it has been further theorized by the same group that combining fibrin glue with autologous blood may allow for a reversal in this flow gradient. In a 2010 study published by Franzini et al. involving 28 patients, 70.4 % (19/27) success rate at 3 months, 81.8 % (18/22) success rate at 1 year, and 83.8 % (10/11) success rate at 3 years were reported (Franzini et al. 2010). A study published in 2013 by Franzini et al. found complete symptom resolution in 87 % of an 80 patient cohort at 3 months after a single injection (Franzini et al. 2013). Those who had not responded received a second epidural injection and collectively a success rate of 90 % was reportedly encountered.

The patients in our study did not achieve nearly the same level of success, although a subset of the patients did appear to receive substantial benefit from the combined fibrin glue-blood patching procedure. While no patients had complete resolution of their headache 1 week after the injection, 3 of the 8 patients (37.5 %) did report some pain relief at 7 days. At 3-month follow-up, only 1 patient (12.5 %) had complete resolution of her headache, while an additional 3 (37.5 %) had prolonged partial resolution of their symptoms. Unfortunately, 1 patient (12.5 %) had prolonged increased pain after the procedure that persisted at 3-month follow-up. While our results were not as encouraging as those of prior reports, they do indicate that the combined blood and fibrin glue patch may prove beneficial in a subset of medically refractory patients who have failed conservative treatments and conventional EBPs.

The small sample size is a limitation of our study. A study involving a larger number of patients is underway. Of significant note, we were not able to reproduce the exact procedural protocol of prior original studies (Franzini et al. 2010) in which 5 mL autologous blood, 5 mL fibrin glue and 3 mL Isovue were described as being combined within a single syringe prior to injection into the epidural space at L1—2. We found that contact between blood and fibrin glue in a single syringe caused fairly immediate clotting with formation of a pasty compound, which precluded further injection. To overcome this procedural limitation we modified the protocol to allow injection of fibrin glue first, followed by an injection of autologous blood mixed with contrast. This resulted in a similar total volume and fibrin glue-to-blood ratio.

## Conclusions

We noted some benefit for some patients from the injection of both fibrin glue and autologous blood into the epidural space. We are hesitant to advocate the regular use of this technique for all patients with SIH headaches because of the expense, efficacy, and possibility of side effects of this technique in the setting of only modest expected efficacy. However, this procedure may prove to be useful in refractory cases. A pre-procedure discussion of the risks and benefits is warranted.

## Abbreviations

CSF: cerebrospinal fluid
CTM: computed tomography myelography
EBP: epidural blood patch
GdM: gadolinium myelography
MRI: magnetic resonance imaging
NRS: numeric rating scale
SEM: standard error of the mean
SIH: spontaneous intracranial hypotension
