# Erratum to: Efficacy of epidural blood patch with fibrin glue additive in refractory headache due to intracranial hypotension: preliminary report

**DOI:** 10.1186/s40064-016-2451-7

**Published:** 2016-07-04

**Authors:** Justin J. Elwood, Misha Dewan, Jolene M. Smith, Bahram Mokri, William D. Mauck, Jason S. Eldrige

**Affiliations:** Department of Anesthesiology, Mayo Clinic, 200 First St SW, Rochester, MN 55905 USA; S.E. PA Pain Management, 721 Dresher Road, Suite 2500, Horsham, PA 19044 USA; Pain Specialists of Iowa, 12499 University Ave, Suite 280, Clive, IA 50325 USA; Department of Neurology, Mayo Clinic, 200 First St SW, Rochester, MN 55905 USA

## Erratum to: SpringerPlus (2016) 5:317 DOI 10.1186/s40064-016-1975-1

In the publication of this article Elwood et al. (2016), the middle initials of the authors were not included in the author list. This has now been corrected in the original article.
